# Deciphering fact from artifact when using reporter assays to investigate the roles of host factors on L1 retrotransposition

**DOI:** 10.1186/s13100-016-0079-3

**Published:** 2016-11-22

**Authors:** Pamela R. Cook, G. Travis Tabor

**Affiliations:** 1 Laboratory of Cell and Molecular Biology, National Institute of Diabetes and Digestive and Kidney Diseases, National Institutes of Health, 8 Center Drive, Bethesda, MD 20892 USA; 2 National Institute of Child Health and Human Development, National Institutes of Health, 35 Convent Drive, Bethesda, MD 20892 USA

**Keywords:** L1, LINE-1, Reporter, Host factor, p38, HSV-TK, SV40, Promoter, Renilla

## Abstract

**Background:**

The Long INterspersed Element-1 (L1, LINE-1) is the only autonomous mobile DNA element in humans and has generated as much as half of the genome. Due to increasing clinical interest in the roles of L1 in cancer, embryogenesis and neuronal development, it has become a priority to understand L1-host interactions and identify host factors required for its activity. Apropos to this, we recently reported that L1 retrotransposition in HeLa cells requires phosphorylation of the L1 protein ORF1p at motifs targeted by host cell proline-directed protein kinases (PDPKs), which include the family of mitogen-activated protein kinases (MAPKs). Using two engineered L1 reporter assays, we continued our investigation into the roles of MAPKs in L1 activity.

**Results:**

We found that the MAPK p38δ phosphorylated ORF1p on three of its four PDPK motifs required for L1 activity. In addition, we found that a constitutively active p38δ mutant appeared to promote L1 retrotransposition in HeLa cells. However, despite the consistency of these findings with our earlier work, we identified some technical concerns regarding the experimental methodology. Specifically, we found that exogenous expression of p38δ appeared to affect at least one heterologous promoter in an engineered L1 reporter, as well as generate opposing effects on two different reporters. We also show that two commercially available non-targeting control (NTC) siRNAs elicit drastically different effects on the apparent retrotransposition reported by both L1 assays, which raises concerns about the use of NTCs as normalizing controls.

**Conclusions:**

Engineered L1 reporter assays have been invaluable for determining the functions and critical residues of L1 open reading frames, as well as elucidating many aspects of L1 replication. However, our results suggest that caution is required when interpreting data obtained from L1 reporters used in conjunction with exogenous gene expression or siRNA.

## Background

The only active, autonomous mobile DNA element in humans is the Long INterspersed Element-1 (LINE-1, L1) retrotransposon, which is responsible for generating almost half of the human genome via insertion of its own DNA and that of non-autonomous Short-INterspersed repeat Elements (SINES) [1]. These insertions, combined with 3′ transductions, nonallelic homologous recombination and mobilization of cellular mRNAs, have had a defining impact on genomic architecture, and the consequences on gene regulation and human development are largely unknown [2–5]. L1 activity is restricted to certain cell types (reviewed in [6]), and retrotransposition is thought to occur mainly in embryonic cells [7, 8], pluripotent stem cells [9, 10], adult neuronal development [11–15], and cancer [16–19]. Clinical interest in L1 has increased due to its mutagenic and disease-causing potential [11, 20–23], as well as its association with cancer [16–19]. In addition, a growing number of studies suggest that transposable elements can be co-opted to serve fundamental physiological functions [24–30]. Recent work has thus been aimed at identifying cellular host factors required for L1 expression, repression and reactivation. With respect to this, our laboratory recently demonstrated that host proline-directed protein kinase (s) (PDPKs) phosphorylate the L1 protein ORF1p on multiple PDPK motifs required for L1 retrotransposition [31].

PDPK target motifs consist of serines or threonines with a proline in the +1 position (S/T-P motifs), which in ORF1p are: S18/P19; S27/P28; T203/P204; and T213/P214. The PDPK family includes mitogen-activated protein kinases (MAPKs), cyclin dependent kinases (CDKs) and glycogen synthase kinase 3 (GSK3). Prior to our finding that the phosphorylation of ORF1p by PDPKs is necessary for L1 activity, several studies reported associations between L1 and the PDPK p38 [32–34], a MAPK that exists in four different isoforms, α, β, γ and δ [35]. Moreover, the expression of one isoform, p38δ, can be induced in primary cell cultures via exogenous expression of ORF1p [34].

Given these associations between L1 and the PDPK p38, as well as our previous findings that host PDPKs are required for L1 retrotransposition, we decided to investigate the role of each p38 isoform on ORF1p phosphorylation and L1 activity. Although our studies are ongoing, we believe that dissemination of our present findings and their associated experimental pitfalls will be useful to the L1 research community. We report here that: 1) different populations of HeLa cells can result in different experimental outcomes; 2) two presumably complementary L1 retrotransposition reporter assays produced conflicting results when coupled with exogenously expressed p38δ; and 3) two different non-targeting control (NTC) small interfering RNA (siRNA) sequences differentially affected measured L1 activity.

## Results

### MAPK p38δ phosphorylates ORF1p on S/T-P motifs

**Fig. 1:**
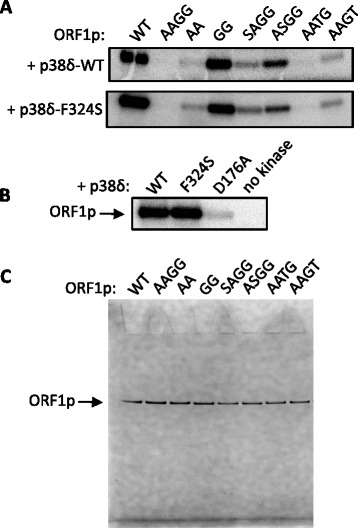
The MAPK p38δ phosphorylates ORF1p on S/T-P motifs required for L1 retrotransposition. **a** ORF1p-WT or S/T-P mutants (200 μM), purified from *E. coli*, were incubated with 85 nM activated p38δ-WT (*top*) or the constitutively active p38δ mutant F324S (*bottom*) in the presence of [γ-^32^P]-ATP; bands on autoradiogram show ^32^P incorporation into ORF1p. ORF1p mutants are S18A/S27A/T203G/T213G (AAGG), S18A/S27A (AA), T203G/T213G (GG), S27A/T203G/T213G (SAGG), S18A/T203G/T213G (ASGG), S18A/S27A/T213G (AATG) and S18A/S27A/T203G (AAGT). **b** ORF1p-WT was incubated with activated p38δ-WT, p38δ-F324S, an inactive p38δ mutant D176A, or no kinase in reactions as described in (**a**). **c** A Coomassie-stained gel shows each ORF1p construct (approximately 100 ng) purified from *E. coli.*

We first determined whether activated wild type p38δ (WT, Invitrogen) could phosphorylate ORF1p on its S/T-P motifs, which are required for robust L1 activity [31]. In vitro radioactive kinase assays revealed that p38δ-WT exclusively phosphorylated bacterially purified ORF1p on these residues, as an ORF1p carrying mutations at all four motifs, S18A/S27A/T203G/T213G (AAGG), was not phosphorylated (Fig. 1a top). We next tested the ability of p38δ-WT to phosphorylate the ORF1p mutants S18A/S27A (AA) and T203G/T213G (GG), and found that the majority of phosphorylation occurred on the GG mutant, which retained both serine motifs (Fig. 1a top).

In order to compare the degree of phosphorylation at each motif, we constructed a series of mutants, each bearing only one intact S/T-P motif: SAGG (S27A/T203G/T213G); ASGG (S18A/T203G/T213G); AATG (S18A/S27A/T213G); and AAGT (S18A/S27A/T203G). S27 (ASGG) was phosphorylated by p38δ-WT to the greatest extent (Fig. 1a top). T213 (AAGT) was phosphorylated to approximately the same degree as S18 (SAGG), but p38δ-WT showed almost no activity on T203 (AATG). Of note, results from the kinase prediction program NetPhosK 1.0 [36] indicated that unspecified p38 isoforms were expected to target ORF1p at S18, T203 and T213, but not S27.

### Constitutively active p38δ-F324S retains ORF1p substrate specificity

Various p38δ mutants that retain some degree of constitutive activity independent of phosphorylation by their activating upstream kinases in the MAPK pathway have been described [37]. In those studies, the constitutively active mutant p38δ-F324S retained the substrate specificity of activated p38δ-WT for glutathione S-transferase activating transcription factor 2 (GST-ATF2) in vitro when p38δ-F324S was purified from bacteria or immunoprecipitated from HEK293 cell lysates. We found that bacterially purified p38δ-F324S also exhibited wild type substrate specificity for ORF1p’s S/T-P motifs (Fig. 1a bottom). In addition, we tested the mutant p38δ-D176A, which was reported to have no activity on GST-ATF2 when purified from bacteria but greater activity than p38δ-WT when immunoprecipitated from HEK293 cells [37]. Bacterially purified p38δ-D176A barely phosphorylated ORF1p in vitro compared to p38δ-WT or p38δ-F324S (Fig. 1b). Figure 1c shows each ORF1p construct, purified from *E. coli*, used for the in vitro kinase assays.

### L1 reporter assays

**Fig. 2:**
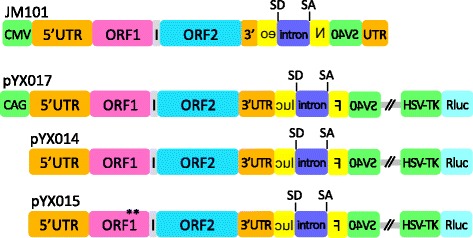
Schematic of L1 reporter plasmids. All reporters contain a full-length L1 element with 5′ and 3′ UTRs (*orange*), ORF1 (*pink*), intergenic region (*gray*), ORF2 (*blue*) and a retrotransposition reporter (*yellow*) interrupted by an artificial intron (*purple*) with splice donor (SD) and acceptor (SA) sites. In JM101, L1 is driven by the CMV promoter (*green*), and in pYX017 by the hybrid CAG promoter (*green*). pYX014 contains only the native L1 promoter in the 5′UTR, and pYX015 is identical to pYX014 except for two missense mutations (R261A/R262A) [38] in ORF1p, rendering pYX015 incompetent for retrotransposition. The reporter in JM101 is an *mneo* cassette driven by the SV40 promoter (*green*) located within the 3′ UTR. The pYX017, pYX014 and pYX015 constructs contain a Firefly luciferase reporter (Fluc), also driven by SV40 (*green*), as well as a gene for Renilla luciferase (Rluc; aqua) driven by the HSV-TK promoter (*green*)

Given our findings that p38δ specifically phosphorylated ORF1p S/T-P motifs, we proceeded to determine the effect of p38δ on L1 retrotransposition. To assess this, we used two previously characterized L1 reporter assays. The original L1 retrotransposition reporter, JM101 (a kind gift from Dr. John Moran), relies on the splicing of an artificial intron from an L1-borne neomycin-resistant gene and its L1-mediated conversion into genomic DNA to produce cell foci resistant to the neomycin analog G418 [38]. Specifically, the reporter contains a full-length L1 element driven by the cytomegalovirus (CMV) promoter and an *mneo* cassette that encodes the neomycin-resistant gene (*neo*), driven by a Simian virus 40 (SV40) promoter located within the 3′ untranslated region (UTR) of L1 (Fig. 2 top). The *neo* gene product, also known as aminoglycoside 3′-phosphotransferase-II (APH (3′)-II), phosphorylates and thereby inactivates G418. Selection with G418 is begun approximately three days following transfection of the reporter plasmid into retrotransposition-competent cells and is continued for 10–12 days. The arrangement of the *neo* gene in JM101 ensures that only cells that have undergone retrotransposition by the L1 reporter element will express APH (3′)-II. The coding sequence for *neo* and its promoter are located on the antisense strand in the 3′ untranslated region of L1. Within this sequence is the engineered artificial intron, but it can only be spliced from the L1 sense RNA driven by the L1 promoter due to the orientation of the splice donor (SD) and splice acceptor (SA) sequences. Once spliced, the L1 RNA is retrotransposed into cDNA and inserted into the genome. After synthesis of the complementary DNA strand, which contains the spliced *neo* template, the transcript for APH (3′)-II can be initiated from the antisense promoter.

The more recently developed single-vector dual luciferase L1 reporters (kind gifts from Dr. Wenfeng An) are based on the same principle as the *mneo* reporter, but instead of *neo* they contain the gene for Firefly luciferase (Fluc). Fluc is also driven by an SV40 promoter and interrupted by an intron to monitor retrotransposition (Fig. 2, lower schematics) [39]. In addition, this reporter contains an internal control gene expressing Renilla luciferase (Rluc) driven by a Herpes simplex virus thymidine kinase (HSV-TK) promoter. Constitutively active Rluc expression is intended as a normalizing control for variations in cell plating, transfection efficiency and survival. Four days following transfection, cells are lysed and retrotransposition is reported as a function of Rluc-normalized Fluc luminescence. The three single-vector luciferase reporters used in this study were: pYX017, which contains an L1 element driven by a hybrid CAG promoter consisting of the CMV enhancer fused with a modified chicken beta-actin promoter and a splice element from the rabbit beta-globin gene [40]; pYX014, which contains only the native L1 promoter in the 5′UTR; and pYX015, a negative control, which is identical to pYX014 except that it carries two missense mutations in ORF1p and is thus retrotransposition-incompetent [38, 39].

### Constitutively active p38δ increases G418-resistant colonies

**Fig. 3:**
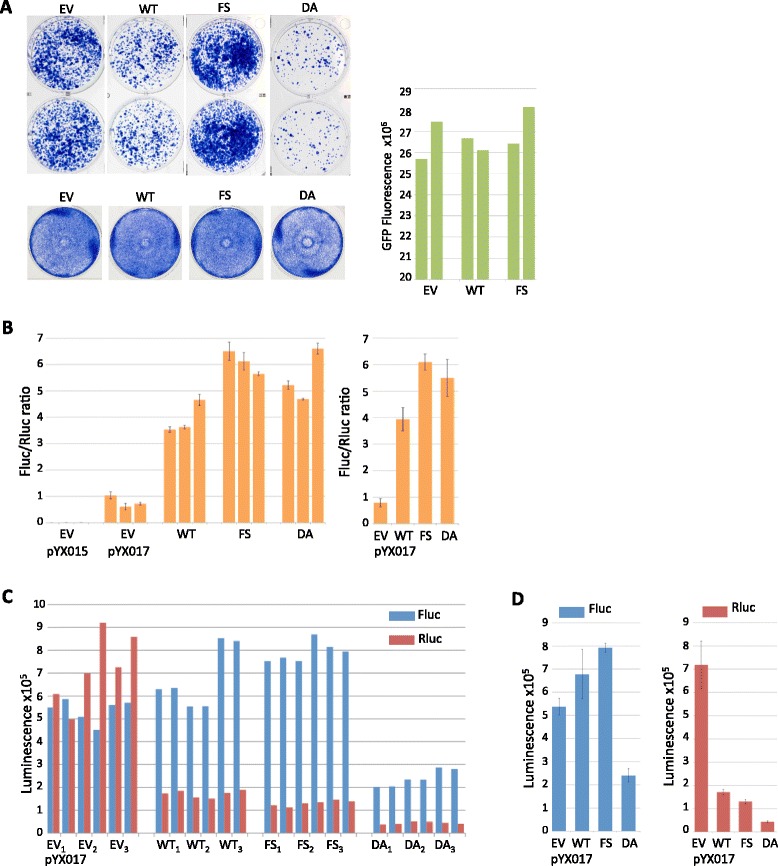
Effects of p38δ on two different L1 reporter assays. **a** *Top rows* show duplicate wells of Giemsa-stained G418-resistant colonies resulting from transfection of the L1 reporter JM101 in the presence of pcDNA mammalian expression vectors for: empty vector (EV), p38δ-WT (WT), p38δ-F324S (FS) or p38δ-D176A (DA). Bottom row shows the effect of each pcDNA expression vector on cell growth. The right panel indicates fluorescence intensities obtained from cotransfection of EGFP with each indicated p38δ construct or empty vector; results from duplicate wells are shown. **b** Relative Fluc/Rluc luminescence ratios obtained from lysates of HeLa cells transfected with the L1 reporter plasmid pYX015 or pYX017 in the presence of indicated pcDNA mammalian expression vectors. Three biological replicates are shown for each experimental condition; error bars represent the SEM from two technical replicates (defined as two distinct samples taken from each biological sample). The graph at right shows the average of three biological replicates shown separately in the left panel; error bars indicate the SEM, *n* = 3 biological replicates. **c** Individual luminescence values are shown for Fluc (*blue*) and Rluc (*red*) used to calculate the Fluc/Rluc ratios from pYX017 in (**b**); technical replicates are side-by-side; biological replicates are indicated in subscript. **d** Mean Fluc and Rluc luminescence values were derived by first averaging the technical replicates for each biological sample (*n* = 2), and then averaging the resulting values of each biological replicate; error bars represent the SEM of biological replicates, *n* = 3

Consistent with our in vitro results and our previous findings that the phosphorylation of ORF1p S/T-P motifs is required for robust L1 activity, we found that exogenous expression of the constitutively active p38δ-F324S (FS) appeared to increase L1 retrotransposition in the G418-based assay relative to an empty vector control (EV), while p38δ-D176A (DA), which failed to appreciably phosphorylate ORF1p in vitro, inhibited L1 (Fig. 3a top). Surprisingly, p38δ-WT (WT) also repressed formation of G418-resistant colonies (Fig. 3a top left). These effects did not appear to be a result of altered cell viability, as only p38δ-D176A somewhat affected cell growth (Fig. 3a bottom left). To determine whether the observed decrease in colony density resulting from p38δ-WT overexpression might be due to effects of the expression vector on cotransfection efficiencies, we cotransfected an expression plasmid for the enhanced green fluorescent protein (EGFP, a kind gift from Dr. Birong Shen) with either the pcDNA empty vector, p38δ-WT or p38δ-F324S. Neither p38δ-WT nor p38δ-F324S appreciably altered EGFP fluorescence compared to the empty vector (Fig. 3a right).

The inhibition of L1 by p38δ-WT may be explained by the fact that p38, like other MAPKs, relies on a complex network of docking interactions with many proteins, including substrates, upstream activating MAPK kinases, phosphatases and scaffolding and regulatory factors. These interactions collectively synchronize the activation and localization of p38 via feedback loops and crosstalk with other pathways [[41] and references therein]. Thus, a pool of excess, unactivated p38δ-WT could perturb this regulatory system, or may simply compete with the population of endogenous activated p38, resulting in inhibition of L1. Consistent with this possibility are several studies that showed expression of a nonfunctional p38 has a dominant negative effect on endogenous p38 activity [42–46]. In addition, during some of our own preliminary experiments, we found on rare occasion that exogenous p38δ-WT slightly increased rather than decreased the number of G418-resistant colonies (unpublished data), further suggesting that the effect of exogenous p38δ-WT could depend on cellular conditions that affect the p38 pathway. For example, confluent stock cultures, as opposed to proliferating cultures, have been found to activate endogenous p38α, with effects lasting up to 48 h after re-plating [47]. However, our investigation of this and several other routine tissue culture variables, including the amount of time cells were exposed to trypsin during sub-culturing, the presence or absence of antibiotics in culture media, lot-to-lot variations in fetal bovine serum (FBS), passage number or overall time in culture, revealed no correlation with the effect of exogenous p38δ on L1 activity (unpublished data). A previous report indicated that individual HeLa clones can exhibit varying degrees of retrotransposition activity and that certain clones may grow to dominate a mixed culture over time [48]. This phenomenon may also bear on how exogenous host factors impact L1 activity.

### Effects of MAPK p38δ-WT differ depending on the L1 reporter assay used

As part of our efforts to understand the effects of p38δ-WT on L1, we used the single-vector dual luciferase assay in parallel with the G418-based assay (i.e. cells were plated from a common suspension and transfected simultaneously using the same reagents). Data from dual luciferase assays are typically normalized to Rluc expression and reported as a ratio of Fluc/Rluc luminescence. Using this method in an experiment done in parallel with the G418-based assay in Fig. 3a, we found that p38δ-WT, p38δ-F324S, and, surprisingly, p38δ-D176A, increased L1 retrotransposition by 5, 7.7 and 7 fold, respectively (Fig. 3b). However, the Fluc/Rluc luminescence ratio is valid only if the expression of Rluc is independent of the experimental treatment. It is obvious from the individual luminescence data for Fluc and Rluc shown in Figs. 3c and d that p38δ expression dramatically affected Rluc luminescence. Such a decrease in Rluc in the absence of a corresponding decrease in cell survival or transfection efficiency would thus artificially inflate the Fluc/Rluc ratio. As shown previously, cell growth was not detectably affected by p38δ-WT or p38δ-F324S, and we detected no differences in cell densities in any wells during the course of the luciferase assay. Moreover, we found no effect from p38δ-WT or p38δ-F324S in the previous cotransfection efficiency control experiment using EGFP. Combined, these data strongly suggest that Rluc, driven by the HSV-TK promoter, is an inadequate normalizing control for these experiments.

Rluc expression notwithstanding, Fluc, like APH (3′)-II, reports on raw retrotransposition events and would thus be expected to produce results paralleling those of the G418 assay under similar experimental conditions. If we then consider only Fluc luminescence, the effects of p38δ-F324S and p38δ-D176 roughly coincide in direction if not degree with those observed in the G418 assay. However, p38δ-WT appears to affect the two reporters differently, inhibiting G418-resistant colony formation but slightly increasing Fluc luminescence (Figs. 3a, c and d left). As with the G418 assay, our preliminary experiments using the dual luciferase assay sometimes showed an outlier effect of p38δ-WT, but in this case the outlier was repression of Fluc (unpublished data). Although sub clonal HeLa populations may have been a contributing factor in those experiments, which utilized different stocks of cells, it would not explain differential effects of p38δ-WT on two reporters in experiments performed in parallel using a common suspension of HeLa cells.

Two questions thus arose: 1) why did p38δ-WT predominantly decrease colony numbers in the G418 assay but increase Fluc luminescence, while the effects of p38δ-F324S and p38δ-D176A remained consistent between the two reporters, and 2) what is the cause of decreased Rluc expression in the presence of p38δ?

With respect to the first question, it may be significant that variations were most evident in response to p38δ-WT since it, unlike F324S, would be dependent on a network of cellular factors for activation. This possibility notwithstanding, if the inhibitory effects of p38δ-WT in the G418-based assay arose from competition with endogenous p38δ, one would expect equivalent competition, not activation, with the pYX017 reporter. Since this was not what we observed, we then considered variables in the assays themselves that might explain the differential effects of p38δ-WT.

**Fig. 4:**
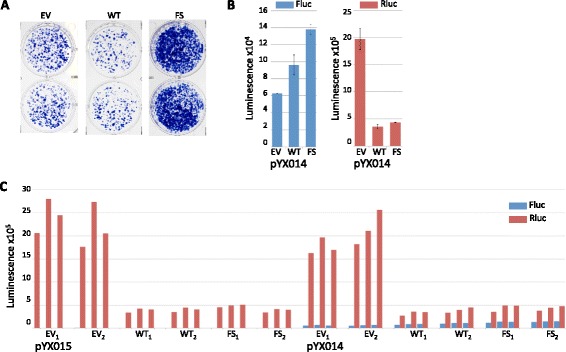
p38δ increases Fluc independent of a heterologous promoter. **a** Duplicate wells containing G418-resistant colonies resulting from transfection of HeLa cells with the L1 reporter JM101 in the presence of pcDNA mammalian expression vectors for: empty vector (EV), p38δ-WT (WT) or p38δ-F3324S (FS). **b** Mean Fluc (*left*) and Rluc (*right*) luminescence values obtained from lysates of HeLa cells transfected with the L1 reporter plasmid pYX014 in the presence of indicated pcDNA mammalian expression vectors. Averages were derived from raw data shown in (**c**) by first averaging technical replicates for each biological sample (*n* = 3), and averaging biological replicates; error bars represent SEM of biological samples, *n* = 2. **c** Individual luminescence values are shown for Fluc (*blue*) and Rluc (*red*) used to calculate averages in (**b**); technical replicates are side-by-side; biological replicates are indicated with subscripts

The first and most obvious difference between the two reporters is that L1 is driven by a CMV promoter in JM101 but a CAG promoter in pYX017, though the CAG promoter contains a CMV enhancer element (Fig. 2). CMV promoters can be affected by some p38 isoforms [49–53], but we did not observe a significant effect of p38δ-WT or p38δ-F324S on EGFP, which is also driven by a CMV promoter. To address whether the increase in Fluc luminescence stemmed from effects of p38δ on the CAG promoter, we used the pYX014 construct, which is identical to pYX017 except that it relies on the native L1 promoter in the 5′ UTR for L1 expression instead of CAG (Fig. 2). Using JM101 in parallel with pYX014, we again found that p38δ-WT inhibited formation of G418-resistant colonies (Fig. 4a), while both p38δ-WT and p38δ-F324S increased Fluc luminescence from pYX014 by 1.5 and 2.2 fold, respectively (Figs. 4b left and c), compared to 1.3 and 1.5 fold from pYX017 (Fig. 3d left). Since p38δ-WT increased Fluc in both pYX014 and pYX017, the effect of p38δ-WT appears to be independent of the CAG promoter in pYX017. We eliminated p38δ-D176A from this and further experiments given its effect on cell growth (Fig. 3) as well as the report that, despite its inactivity in vitro, it can be activated in HEK293 cells [37], making its effects on L1 uninterpretable, particularly given the inhibitory effect of p38δ-WT on G418-resistant colony formation.

Regarding the effect of p38δ on Rluc luminescence, we considered three possible explanations: 1) cell death; 2) transcription or translation interference from pcDNA-p38δ; or 3) inhibition of the Rluc HSV-TK promoter.

As stated earlier, we found no evidence of cell death, despite a 76–94% decrease in Rluc luminescence using pYX017 (Figs. 3c and d right) and similar decreases with pYX014 (Fig. 4b right and c). Moreover, the decrease in Rluc luminescence from the retrotransposition defective pYX015 (Fig. 4c) ruled out the possibility that rampant L1 activity severely compromised the cells, an event the G418-based assay could have potentially missed.

**Fig. 5:**
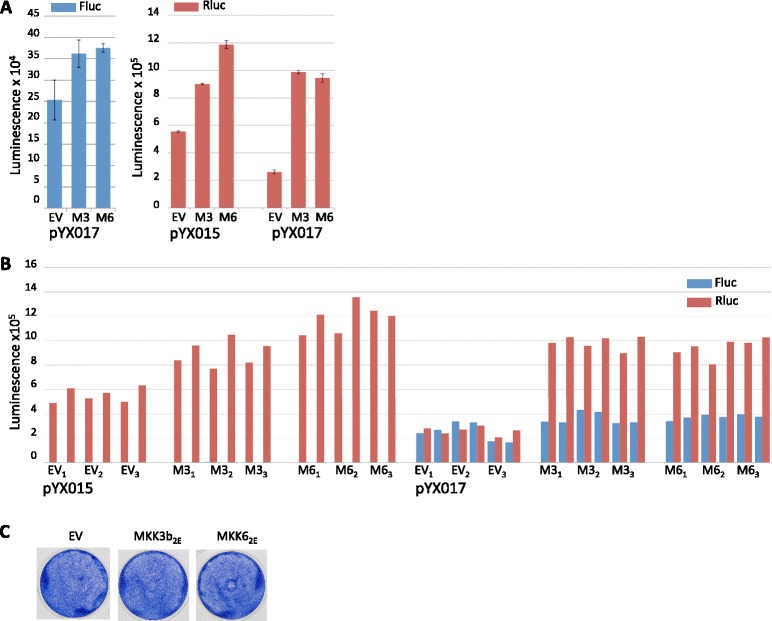
MKK3b_2E_ and pcDNA-MKK6_2E_ increase Rluc luminescence. **a** Mean Fluc (*left*) and Rluc (*right*) luminescence values obtained from lysates of HeLa cells transfected with the L1 reporter plasmid pYX015 or pYX017 in the presence of pcDNA-MKK3b_2E_ (M3) or pcDNA-MKK6_2E_ (M6). Averages were derived from data shown in (**b**) by first averaging technical replicates for each biological sample (*n* = 2), then using this value to average biological replicates; error bars represent SEM of biological samples, *n* = 3. **b** Individual luminescence values are shown for Fluc (*blue*) and Rluc (*red*) obtained from lysates transfected with pYX015 or pYX017 and the indicated pcDNA expression vectors; technical replicates are side-by-side; biological replicates are indicated with subscripts. **c** Wells show effects on cell growth in response to expression of pcDNA-MKK3b_2E_ (M3) or pcDNA-MKK6_2E_ (M6)

The second option was that decreased Rluc luminescence resulted from generalized transcription and/or translation interference from the cotransfected plasmids. Competition for cellular factors can be relevant at multiple points, including promoter binding, transcription initiation, elongation or translation [54–57]. For example, the different levels of Rluc luminescence from pYX017 (Fig. 3) compared with pYX014 (Fig. 4) might suggest that the highly active heterologous CAG promoter in pYX017 competed with factors required by the HSV-TK promoter driving Rluc in pYX017. Also, the empty vector control lacked an optimized Kozak sequence, which may have rendered it less effective at competing for translational machinery than the p38δ constructs. To determine if the kinase-containing plasmids competed with pYX017 for factors necessary for Rluc expression, we cotransfected the L1 reporter with plasmids encoding constitutively active MAPK-kinases (MAPKKs) MKK3b-S288E/T222E (M3) or MKK6-S207E/T211E (M6), which are specific upstream activators of p38 isoforms [58–60]. Unlike p38δ, each MKK upregulated Rluc (Fig. 5a right and b). As expected, each MKK also increased Fluc (Fig. 5a left), presumably via activation of an endogenous p38. Neither of the MKKs had any effect on cell growth (Fig. 5c). These results strongly suggest that inhibition of Rluc by p38δ is a specific rather than indiscriminate effect.

The ability of p38δ to inhibit the Rluc HSV-TK promoter was not empirically determined by us, but multiple reports show that HSV-TK promoters, including those driving Renilla, can be perturbed by multiple experimental conditions [61–64]. These include the expression of the Sp1 transcription factor [64], which is upregulated by p38 [65]. We consider the potential effects of p38 on the HSV-TK and SV40 heterologous promoters, as well as other elements of the L1 reporters, in greater detail in the discussion.

### Two non targeting control siRNAs differentially affect reported L1 activity

**Fig. 6:**
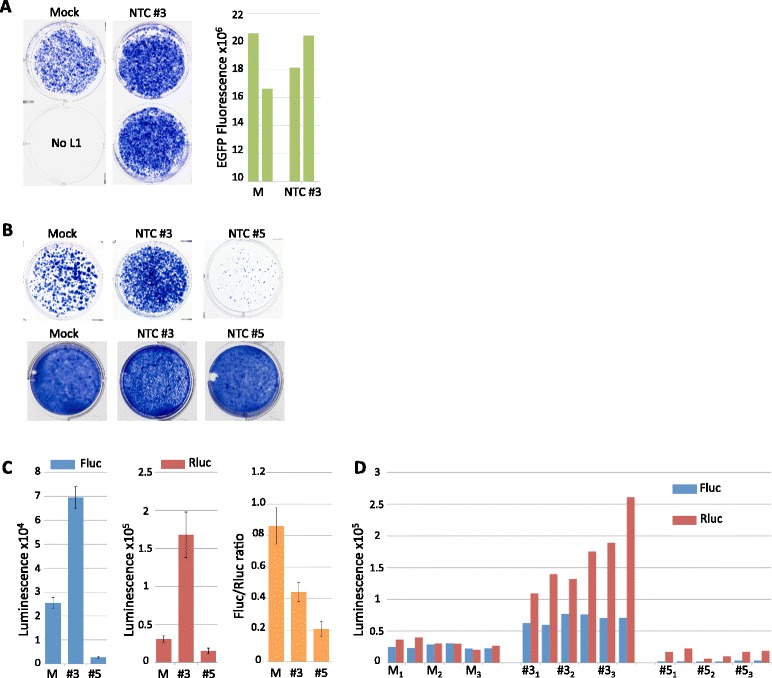
NTC siRNAs have differential effects on L1 reporter assays. **a** Wells show G418-resistant colonies resulting from transfection of the L1 reporter JM101 in the presence of no siRNA (mock, with transfection reagent only) or 10 nM NTC #3 siRNA. Graph at right shows EGFP fluorescence from cells pretreated with 10 nM NTC #3 siRNA or mock (M); results from duplicate wells are shown. **b** *Top row* shows G418-resistant colonies resulting from the transfection of the L1 reporter JM101 in the presence or absence of 25 nM of indicated siRNA; bottom row shows effect of 25 nM of indicated siRNA on cell growth. **c** Mean Fluc (*left*) and Rluc (*second from right*) luminescence values obtained from lysates of HeLa cells transfected with the L1 reporter pYX017 in the presence of no siRNA (M) or 25 nM NTC #3 or NTC #5; averages were derived from data shown in (**d**) by first averaging technical replicates for each biological sample (*n* = 2), then using this value to average biological replicates; error bars represent SEM of biological samples, *n* = 3; average Fluc/Rluc ratios (*third from right*) are also shown. **d** Individual luminescence values are shown for Fluc (*blue*) and Rluc (*red*) obtained from lysates of HeLa cells transfected with pYX017 and the indicated siRNA; technical replicates are side-by-side; biological replicates are indicated with subscripts

While investigating the effect of p38δ on L1 retrotransposition, we performed siRNA experiments using a SMARTpool mixture against p38δ (Dharmacon, M-003591-02-0005) and the NTC siRNA #3 (Dharmacon). Although the siRNA against p38δ dramatically reduced the number of G418-resistant colonies relative to NTC #3, RT-PCR showed no significant knockdown of the p38δ transcript (data not shown). Interestingly, however, NTC #3 considerably increased colony density relative to the mock control (Fig. 6a left). EGFP fluorescence from cells pretreated with siRNA prior to transfection suggested that the siRNA had little impact on transfection efficiency (Fig. 6a right). Given these unexpected results, we tested an additional control siRNA, NTC #5, also from Dharmacon. In marked contrast to NTC #3, NTC #5 dramatically reduced G418-resistant colonies relative to the mock control (Fig. 6b top). Neither NTC dramatically affected cell growth, though NTC #3 had a slight inhibitory effect (Fig. 6b bottom). It is notable that unlike p38δ-WT, the NTC siRNAs exerted their respective effects similarly on both Fluc luminescence and G418-resistant colony formation (Fig. 6b top, c left and d). However, L1 activity as reported by the Fluc/Rluc ratio appears to be decreased by NTC #3 rather than increased (Fig. 6c). We did not further investigate potential causes for these results. Information on Dharmacon’s website states that each NTC is reported to contain a minimum of 4 mismatches to all human, mouse and rat genes and to have minimal effects in genome-wide targeting via microarray analyses. We did not test Dharmacon’s NTC #1, as it was reported to increase cell growth (personal communication, Dharmacon), nor NTC #2 or #4 due to their targeting of Firefly luciferase (Dharmacon website).

## Discussion

Engineered L1 reporter assays have tremendously advanced the field of L1 research, allowing investigators to examine key details of the retrotransposition process [66]. Through mutational analyses, critical amino acids in ORF1p and ORF2p have been identified, leading to a greater understanding of the form and functions of these proteins and their roles in L1 retrotransposition. Investigations of L1 insertion sites, 5′ truncations, untranslated regions, native L1 promoters and the poly (A) tail have all been made possible by these assays, as have numerous comparative evolutionary studies of extinct L1 fossils in the human and mouse genomes. Our own work on the role of ORF1p phosphorylation would not have been possible without these reporters.

Importantly, we have not observed variation in relative differences between an L1-WT control and any L1 mutant in our history of working with L1 reporter plasmids. In other words, any mutant L1 construct we have made consistently exhibits the same degree of change in G418-resistant colonies relative to a WT control within a given experiment, independent of differences in cell populations. Thus, the L1 reporters are particularly reliable for investigating *cis* aspects of L1—the purpose for which the original reporter was designed. However, the results presented here strongly suggest that data derived from L1 reporters when used in conjunction with exogenous gene expression or siRNA to investigate the roles of host factors may be challenging to interpret. Although we have not exhaustively investigated possible factors that would account for our results, we feel these data are nonetheless informative and potentially timesaving for other researchers intending to use these approaches to investigate interactions between L1 and its host.

Our efforts to determine the effect of p38δ on L1 retrotransposition using engineered L1 reporters underscore the complexities inherent in such endeavors. The p38 signaling pathway itself is extremely complex, with different isoforms having unique, overlapping or competing functions depending on the cell type, or even within the same cell under different conditions [35, 67–69]. This complexity is compounded by the possibility that different p38 isoforms may have competing specificities and functional outcomes on ORF1p and other substrates relevant to L1 activity, as well as on heterologous promoters in L1 reporters.

A case in point is the repression of Rluc by exogenous p38δ. Previous reports show that p38 can activate late HSV promoters [70] as well as the transcription factor Sp1 [65], which both binds [71] and activates the HSV-TK promoter [61, 64]. These studies would suggest that if p38δ had an effect on HSV-TK, it would be activation, not repression. However, this assumption would be an oversimplification given the complexity of p38 signaling and reports that p38 isoforms can compete with one other with opposing effects [68]. An alternative possibility is that over-expression of exogenous p38δ perturbed constitutive activation of the HSV-TK promoter by interfering with a different endogenous p38 isoform. This possibility is supported by two observations. First, each p38δ construct repressed Rluc luminescence despite the fact that each has widely varying catalytic activities in vitro as well as different effects on L1 activation. Equivalent effects from each p38δ construct would be expected only if the effect were mediated by something other than their catalytic kinase activity; e.g., competition for docking interactions with limiting regulatory factors required by other p38 isoforms. Second, since MKK3b-2E and MKK6-2E selectively activate only p38 isoforms [72], their activation of Rluc strongly suggests that HSV-TK is indeed activated by an endogenous p38 isoform, but not p38δ. Combined, these data indicate that the ability of both active and inactive exogenous p38δ to repress the HSV-TK promoter derives from competition for host regulatory proteins by another, endogenous p38 isoform.

While most of our focus here has been on possible sources of artifact arising from the single vector dual luciferase assay, p38δ-WT and p38δ-F324S similarly activated Fluc in those assays; it was only in the G418-based assay where contradictory results between p38δ-WT and F324S were observed, with significant inhibition of apparent retrotransposition in response to p38δ-WT but strong activation by F324S. Since p38δ-WT gave conflicting results in these assays, it may be worth discussing potentially relevant variations between the assays.

One notable difference is the lack of the Epstein-Barr nuclear antigen 1 (EBNA1) gene and the Epstein-Barr virus (EBV) origin of replication on the single vector dual luciferase reporters, which were not required due to the shorter experimental time relative to the G418-based assay [39]. EBNA1, however, contains multiple phosphorylation sites required for the maintenance of plasmids and transcriptional activation [73, 74]. Specifically, the EBNA1 nuclear localization sequence contains two S/T-P motifs, whose phosphorylation is required for nuclear import [73–75]. Although at least one of these motifs is thought to be targeted by CDKs [75], it is possible that phosphorylation of one or both S/T-P motifs is perturbed by exogenous p38δ-WT expression via competition for regulatory factors.

Another difference between the two assays is their respective reporter genes. The G418-based assay relies on expression of APH (3′)-II to monitor L1 retrotransposition. However, in addition to inactivating aminoglycosides via phosphorylation, two APH isoforms have also been found to phosphorylate proteins. Although it is not known whether the neomycin resistance gene APH (3′)-II or the hygromycin resistant gene APH (4)-I, also present on JM101, can similarly target cellular proteins, caution has been urged in their use as selectable markers if such activity might interfere with the experimental design [76].

A source of potential artifact for both assays is the SV40 promoter, which drives the *neo* and Fluc reporter cassettes. As noted earlier, p38 is known to activate the transcription factor Sp1, which in addition to binding the HSV-TK promoter also binds and activates the SV40 early promoter [77]. Moreover, the SV40 promoter contains binding sites for AP-1 transcription factors [78, 79], which are activated by the isoform p38β but can be inhibited by p38γ or p38δ [68]. Thus, perturbed expression, in either direction, of an already spliced and integrated Fluc gene could falsely report on retrotransposition events. It is unclear, however, whether an increase above a given baseline expression of APH (3′)-II would alter colony viability or growth during G418 selection. Also of note, a recent study of the effects of heavy metals on L1 found that cobalt increased the activity of the SV40 promoter in HeLa cells but decreased its activity in human fibroblasts and the human neuroblastoma cell line BE (2)-M17 [80], indicating that heterologous promoters can be differentially affected by the same variables in different cell lines. This raises the possibility that different clonal populations of the same cell type might also respond differentially to exogenous factors.

Regarding potential effects arising from the CMV promoter, although p38δ did not appear to affect expression of the CMV-driven EGFP, we imaged EGFP expressing cells 24 h post transfection for the purpose of monitoring transfection efficiencies, whereas G418 selection was begun three days post transfection. Thus, though EGFP appeared to report equivalent transfection efficiencies, it may not have accurately reflected cumulative effects of p38δ on a CMV promoter after 72 h. With respect to transfection efficiency controls, the potential for exogenous factors to impact these reporters remains an issue, as was demonstrated by the effects of p38δ on Rluc luminescence, which is the transfection efficiency reporter for the luciferase assay, versus no effect on from p38δ on EGFP fluorescence, which is also a common reporter for transfection efficiencies in a variety of assays.

The use of siRNA to probe the functions of cellular genes is a common technique, but the potential for off-target effects is a major drawback. This is typically accounted for by using NTC siRNA, with the assumption that NTC and target siRNAs produce equivalent off-target effects. While this may be true for some experimental systems, the dramatically different effects of NTC #3 and NTC #5 on L1 reporter output suggest a potential problem when these methods are used together. First, interpretations regarding the effect of a targeting siRNA based on comparison to a given NTC would be skewed if the siRNAs produced dissimilar off-target artifacts. This is true even if one confirms knockdown of the target gene. For example, if the target siRNA knocks down a gene of interest (GOI) by 50% and decreases L1 retrotransposition by 50%, one might conclude that knocking down the GOI decreases L1 activity if control siRNA #3 was the non-targeting control. In contrast, if one happened to use control siRNA #5, the conclusion would have been the opposite; i.e. that knockdown increased L1 activity.

In addition, it is possible that targeting siRNAs could induce the same types of artifacts we observed with the NTC siRNAs. For example, despite a hypothetical parallel 50% knockdown of the GOI and L1 activity, the decrease in L1 activity may have been due solely to off-target effects unrelated to gene knockdown. Similarly, it may be possible that off-target effects that increase apparent L1 activity could mask a genuine inhibitory effect mediated by gene knockdown. Our data with NTC #3 and #5 show that it is unreliable to control for such off-target effects by using non-targeting control siRNAs alone, as their effects can vary dramatically and may not be equivalent to those induced by targeting siRNAs. The most well- established method for confirming that results from targeting siRNA are due to GOI knockdown is the cotransfection of siRNA-resistant rescue plasmids. However, the interpretation of these results may still be complex in certain situations, as evidenced by our finding that p38δ-WT can both repress and activate L1 activity in different assays and cellular contexts.

In addition, our finding that non-targeting control siRNAs may affect L1 retrotransposition may have relevance not only for interpreting L1 assays but also for the development of therapeutic siRNA, a treatment option currently being optimized for numerous conditions including cancer [81–83]. As L1 is thought to have deleterious effects, caution is warranted in the design and testing of candidate molecules intended for clinical use.

Effects on heterologous promoters can be monitored in order to select one unaffected by experimental conditions. However, as some L1 reporters have up to three such promoters and may also be susceptible to artifacts arising from EBNA1 and the EBV origin of replication, this approach could be costly in terms of labor and resources and is therefore impractical for high throughput screening utilizing multiple experimental conditions. However, assuming suitable promoters could be identified for each experimental condition, a combination of native and constitutive L1 promoters with corresponding assays to monitor cell growth may be employed to successfully identify effects on L1 activity.

Several recently developed methods may offer some alternatives [84, 85]. The L1 element amplification protocol (LEAP assay) allows investigation of in vitro ORF2p enzymatic activity from L1 RNP particles purified from cells expressing engineered L1 reporters [86, 87]. The addition of purified host factors to these reactions would allow investigation of direct effects on ORF2p reverse transcriptase activity while avoiding some of the issues described herein. Next-generation sequencing methods [85, 88] including retrotransposon capture sequencing (RC-seq) [89, 90], as well as novel approaches for validation such as droplet digital PCR [91], offer the possibility of examining endogenous L1 elements in their native chromatin environment. These technical advances should facilitate investigation of the host factors that delimit L1 tissue specificity and various aspects of retrotransposition.

## Conclusions

Our results indicate that the use of exogenous gene expression or siRNA with engineered L1 reporter assays may introduce confounding variables. Thus, investigation of the roles of host factors in L1 retrotransposition when using these techniques will require extra efforts to ensure that observed results are not artifacts.

## Methods

### Plasmid construction

Bacterial expression vectors for ORF1p (pET32aΔN-ORF1-6xHis) were made as follows. First, an existing ORF1 vector [92] with the backbone of pET32a was altered to remove the following: the pET32a N-terminal TRX and 6xHis tags, an engineered TEV sequence that had previously destroyed the multiple cloning region, a truncated ORF1 mutant and remnant sequence 3′ to ORF1 that was retained from prior subcloning. A remaining 3′ EcoRI site and the C-terminal 6xHis tag were left intact, and BamH1 site was inserted 5′ of the EcoRI site. These changes were made using the QuikChange II kit (Agilent) with the forward deletion primer 5′TTAACTTTAAGAAGGAGATATACATGGATCCAATCCCGGGACGCGTG and reverse deletion primer 5′CACGCGTCCCGGGATTGGATCCATGTATATCTCCTTCTTAAAGTTAA. The resulting clone was designated pET32aΔN. Full-length ORF1 PCR-generated amplicons were created from the previously described pORF1-Flag mammalian expression vector [31] using a high-fidelity DNA polymerase with the forward primer 5′CGCGGATCCATGGGGAAAAAACAGAACAG containing a 5′ BamH1 site, and reverse primer 5′GCCGGAATTCGCCGCCGCCCATTTTGGCATGATTTTGC, which introduced a spacer of three glycines between the end of ORF1 and the 3′ EcoRI sequence (the Flag sequence was not retained). The ORF1p amplicon was inserted into pET32aΔN via the BamH1 and EcoRI sites. The BamH1 site was subsequently deleted to move the ATG start site of ORF1 to an optimal distance from the ribosomal binding site in pET32aΔN and destroy an alternate out-of-frame ATG start site that encompassed the 5′G of the BamH1 site. These changes were made using the QuikChange II kit (Agilent) with the forward primer 5′GAAATAATTTTGTTTAACTTTAAGAAGGAGATATACATATGGGGAAAAAACAGAACAG and the reverse primer 5′CTGTTCTGTTTTTTCCCCATATGTATATCTCCTTCTTAAAGTTAAACAAAATTATTTC. In an attempt to reduce translation initiation at internal non-canonical Shine-Dalgarno sequences in ORF1, we also created silent mutations at D123 and N126, changing the existing codons to GAC and AAC, respectively. ORF1p S/T-P motif mutations were created using sequential site-directed mutagenesis with the QuikChange II kit (Agilent).

Bacterial expression plasmids for p38δ-F324S and D176A (pRSET-A-6xHis-p38δ-StrepII) were made by first generating a p38δ-WT amplicon via PCR using a high-fidelity polymerase and the forward primer 5′CGCGGATCCGCAATGAGCCTCATCCGGAAAAAGGGCTTCTACAAGCAGG and reverse primer 5′GCCGGAATTCTCACTTCTCGAACTGGGGGTGGCTCCATGCGCCCAGCTTCATGCCACTCCG on the Addgene template plasmid # 20523 (pWZL Neo Myr Flag MAPK13, a gift from William Hahn & Jean Zhao [93]). The amplicon containing a 5′ BamHI and Kozak sequence and a 3′ Gly/Ala spacer upstream of a StrepII tag, stop codon and EcoRI site was then inserted into pRSET-A (ThermoFisher) via the BamHI and 3′ EcoRI sites in the multiple cloning region. Point mutations were created via site-directed mutagenesis with the QuikChange II kit (Agilent).

The mammalian expression vector for p38δ-WT (pcDNA-Zeo (3.1+)-p38δ-StrepII) was made by PCR amplification of the Addgene plasmid # 20523 [93] using the same forward and reverse primers noted above for making pRSET-A-6xHis-p38δ-StrepII, followed by insertion into the multiple cloning region of pcDNA 3.1/Zeo (+) (ThermoFisher). Point mutations to make F324S and D176A were created via site-directed mutagenesis with the QuikChange II kit (Agilent).

Mammalian expression vectors for MKK3b_2E_ (pcDNA3 Flag MKK3b (Glu) [58]; Addgene plasmid # 50449) and MKK6_2E_ (pcDNA3-Flag MKK6 (Glu) [60]; Addgene plasmid # 13518) were both gifts from Roger Davis.

All cloned inserts were verified with DNA sequencing. DNA intended for cell culture transfections was purified using the endotoxin-free NucleoBond Xtra Midi plasmid DNA purification kit (Macherey-Nagel).

### Protein expression

ORF1p proteins were expressed in Rosetta (DE3) cells (Novagen) transformed with pET32aΔN-ORF1-His. Overnight starter cultures of 15–25 ml LB medium with 100 μg/ml ampicillin and 34 μg/ml chloramphenicol were grown at 37 °C on a rotary shaker at 250 rpm. The following day, cultures were expanded 20 to 50 fold with LB medium containing the indicated antibiotics and grown at 37 °C on a rotary shaker at 250 rpm to an OD_600_ of approximately 0.6. Cultures were then induced with 1 mM isopropyl-beta-D-thiogalactopyranoside (IPTG), grown for an additional 4–6 h, pelleted via centrifugation and frozen at -80 °C. At the time of purification, cells were thawed and resuspended in 5 ml per gram pellet of a buffer containing 100 mM Tris–HCl (pH 8.0), 100 mM NaCl, and 1 mg/ml lysozyme and incubated on ice for 30 min. Following lysozyme digest, lysates were supplemented with 400 mM NaCl (for final concentration of 500 mM), 2 mM dithiothreitol (DTT) and 15 mM imidazole. The lysates were pulled through a 19–21gauge syringe approximately 12 times and centrifuged at 10,000 × g at 4 °C for 20 min. Cleared lysates were applied to Ni-NTA superflow resin (Qiagen) previously equilibrated with lysis buffer (post lysozyme concentrations), rotated for 1 h at 4 °C, washed 4 times with 20 mM Tris–HCl (pH 7.4), 500 mM NaCl, and 25 mM imidazole, then eluted 4 times with 20 mM Tris–HCl (pH 7.4), 500 mM NaCl, 250 mM imidazole, 10% glycerol and 2 mM DTT at a ratio of 1 μl elution buffer per 1 ml of original culture volume. Proteins were dialyzed overnight against 50 mM Tris–HCl (pH 80), 350 mM NaCl, 15 mM KCl, 5 mM MgCl_2_, 20% glycerol, 2 mM DTT, and 1 mM phenylmethylsulfonyl fluoride (PMSF).

p38δ-F324S and p38δ-D176A proteins were expressed in Rosetta2 (DE3) cells (Novagen) transformed with pRSET-A-His-p38δ-StrepII and processed as described above for ORF1p except 150 mM NaCl was used in the dialysis buffers. Note: we found that omission of DTT in the elution and/or dialysis steps of p38δ purification resulted in an inactive protein, consistent with a previous report [94].

All proteins were quantified via denaturing gel electrophoresis with a standard curve of bovine serum albumin followed by staining with Coomassie G-250 PageBlue (ThermoFisher) and analysis with ImageJ [95].

### Kinase assays

In vitro kinase reactions contained 85 nM p38δ or p38δ dialysis buffer and 200 μM ORF1p in 50 mM Tris–HCl (pH 7.4), 10 mM MgCl_2_, 0.1 mM EGTA, 150 mM NaCl, 2 mM DTT, and 2 mM ATP spiked with approximated 0.5 × 10^6^ c.p.m./nmol [γ-^32^P]-ATP (PerkinElmer). Reactions were incubated at 37 °C for 15 min and stopped with the addition of loading buffer supplemented with EDTA to a final concentration of 50 mM. Samples were heated to 98 °C for 10 min then separated via denaturing gel electrophoresis. Gels were dried and exposed using Phosphorimaging.

### Cell culture

HeLa-JVM cells (a kind gift from Dr. John Moran) were cultured in Dulbecco’s Modified Eagle Media (DMEM) with high glucose and pyruvate (Gibco, ThermoFisher) supplemented with 10% Fetal Bovine Serum (Gibco, ThermoFisher, certified heat inactivated, US origin) and 100 Units/ml penicillin and 100 μg/ml streptomycin from a combined formulation (Gibco, ThermoFisher). The cells were maintained at 37 °C in a standard incubator and passaged using 0.05% Trypsin-EDTA (Gibco, ThermoFisher).

### L1 reporter assays

Culture plates were seeded with HeLa-JVM cells in antibiotic-free DMEM with 10% FBS at a density to achieve approximately 50% confluency in 24 h, at which time cells were transfected using a ratio of 3 μl Fugene6 (Promega) per 1 μg DNA. For the G418-based assay, cells were seeded in 6-well plates and transfected with 500 ng JM101 and 500 ng pcDNA per well, allowed to grow for 72 h, then selected with media containing 400 μg/ml G418 sulfate (Geneticin, Gibco, ThermoFisher) for 10–12 days. Cells were then washed with phosphate buffered saline (PBS) and fixed with 2% formaldehyde and 0.2% glutaraldehyde in PBS for at least 30 min at room temperature. Cells were then washed twice with PBS, stained with KaryoMAX Giemsa (Gibco, ThermoFisher) for 1 h at room temperature, rinsed briefly twice with 50% ethanol and then water. For luciferase assays, cells were seeded in 24-well plates and transfected with 200 ng of reporter and 200 ng pcDNA-p38δ per well or 25 ng pcDNA-MKK3b_2E_ or pcDNA-MKK6_2E_. Lysates were harvested 4 days post transfection and processed in 96-well plates with the Dual-Luciferase Reporter Assay System (Promega) according to manufacturer’s protocol.

### Transfection efficiency assays

HeLa-JVM cells were plated in 8-well glass bottom μ-Slides (ibidi GmbH, Martinsried, Germany) in antibiotic-free DMEM with 10% FBS at a density to achieve approximately 60% confluency per well in 24 h. Wells with siRNA were reverse transfected as described in the following section. After a 24-h incubation, cells were transfected as described above with a pcDNA-EGFP expression plasmid (for siRNA wells) or cotransfected with pcDNA-EGFP and each pcDNA-p38δ expression plasmid. The ratio of DNA to surface area was identical to that used in the 6-well plates. After 24 h, cells were rinsed twice with PBS, then DMEM sans phenol red plus 10% FBS was added to each well. Cells were visualized with a Keyence BioRevo BZ-II 9000 digital microscope fitted with a Nikon PlanApo 4×/0.20 objective lens and 49002 ET-EGFP filter set from Chroma (Bellows Falls, VT). Tiled images covering approximately 70% of each well were stitched with Keyence BZ-II Analyzer software, and total fluorescence in each stitched image was quantified in Fiji software using the Integrated Density function.

### siRNA knockdown

HeLa-JVM cells were plated in antibiotic-free DMEM with 10% FBS at a density to achieve approximately 60% confluency in 24 h and reverse transfected per manufactures’ protocol using Lipofectamine RNAiMAX (ThermoFisher) at a ratio of 1 μl RNAiMAX per 8 pmols siRNA. All siRNAs were purchased from Dharmacon: NTC #3, NTC #5 and SMARTpool siRNA against p38δ (Dharmacon, M-003591-02-0005). Following reverse transfection, cells were incubated for 24 h, then siRNA-containing media was removed and replaced with fresh antibiotic-free plating media with 10% FBS at the time of transfection with L1 reporters as described above.

## Abbreviations

APH(3′)-II: Aminoglycoside 3′-phosphotransferase-II
CDKs: Cyclin dependent kinases
CMV: Cytomegalovirus
DTT: Dithiothreitol
EBNA1: Epstein-Barr nuclear antigen 1
EBV: Epstein-Barr virus
EGFP: Enhanced green fluorescent protein
FBS: Fetal bovine serum
Fluc: Firefly luciferase
GSK3: Glycogen synthase kinase 3
GST-ATF2: glutathione S-transferase activating transcription factor 2
HSV-TK: Herpes simplex virus thymidine kinase
IPTG: Isopropyl-beta-D-thiogalactopyranoside
L1, LINE-1: Long INterspersed Element-1
LEAP assay: L1 element amplification protocol
MAPKKs: MAPK-kinases
MAPKs: Mitogen-activated protein kinases
NTC: Non-targeting control
PDPKs: Proline-directed protein kinases
PMSF: Phenylmethylsulfonyl fluoride
RC-seq: Retrotransposon capture sequencing
Rluc: Renilla luciferase
S/T-P: Serine/threonine-proline
SA: Splice acceptor
SD: Splice donor
SINES: Short-INterspersed repeat Elements
siRNA: Small interfering RNA
SV40: Simian virus 40
UTR: Untranslated region
